# Copper-based metal halides for X-ray and photodetection

**DOI:** 10.1007/s12200-022-00048-x

**Published:** 2022-11-21

**Authors:** Fu Qiu, Yutian Lei, Zhiwen Jin

**Affiliations:** School of Physical Science and Technology & Key Laboratory for Magnetism and Magnetic Materials of Ministry of Education, Lanzhou University, Lanzhou, 730000 China

**Keywords:** Copper-based metal halides, X-ray detector, Photodetectors, Scintillators

## Abstract

Copper-based metal halides have become important materials in the field of X-ray and photodetection due to their excellent optical properties, good environmental stability and low toxicity. This review presents the progress of research on crystal structure/morphology, photophysics/optical properties and applications of copper-based metal halides. We also discuss the challenges of copper-based metal halides with a perspective of their future research directions.

**Graphical Abstract:**

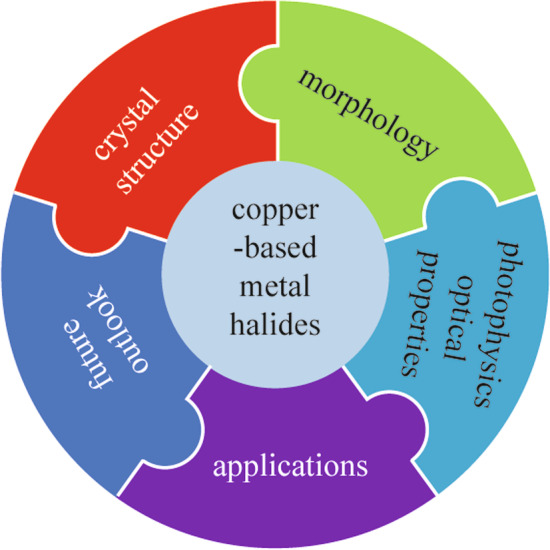

## Introduction

Lead-based metal halide has become a star material in the field of optoelectronic semiconductors due to its excellent optical properties such as high photoluminescence quantum yield (PLQY), tunable emission, and high absorption coefficient [1–5]. Different methods have been used to synthesize all-inorganic metal halides with different morphologies and to study their optical and physical properties. These metal halides have been widely used in solar cells [6–9], LEDs [10–12], photodetectors [13–16], scintillators [17–20] and lasers [21, 22]. However, their further development is hindered by the toxicity of the material.

To overcome the problem of lead (Pb) toxicity, the replacement of metal ions has achieved great success [23, 24]. The same or adjacent main group elements have similar chemical properties to lead, so replacing lead with germanium (Ge) [25], tin (Sn) [26], antimony (Sb) [27], and bismuth (Bi) [28] largely solves the problem of material toxicity. However, Sn^2+^ in Sn-based metal halides is easily oxidized to Sn^4+^ resulting in a significant reduction in the environmental stability of the material [29]. The optical properties of Sb-based and Bi-based metal halides need further enhancement to meet the application requirements [30]. Copper (Cu) is also used in metal ion replacement due to its large reserves, low price and low toxicity, etc.

In 2018, Jun et al. [31] first reported zero-dimensional (0D) Cs_3_Cu_2_I_5_ with low toxicity, ultra-high PLQY, and tunable band gap. Low-dimensional copper-based metal halides are attracting attention due to high natural abundance, excellent photoelectric properties, and low toxicity. Compared with the classical three-dimensional (3D) CsPbX_3_, the low-dimensional copper-based metal halides possess higher exciton binding energy and large Stokes shifts, and ultra-high PLQY; these properties make these materials promising in the field of X-ray and photodetection.

Here, we summarize the development status and existing problems of copper-based metal halides in terms of crystal structure, material morphology, physical properties, optical properties, and applications. At the end of this review, we discuss the challenges of all-inorganic copper-based metal halides and the prospects for future research directions.

## Crystal structure and morphology

### Crystal structure

**Fig. 1:**
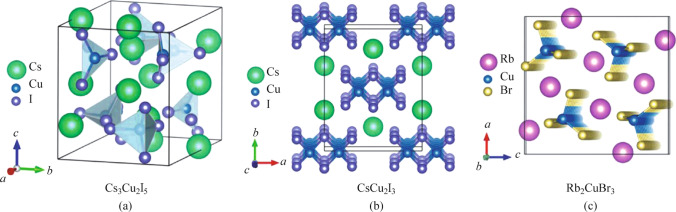
Crystal structures of copper-based metal halides: **a** Cs_3_Cu_2_I_5_; **b** CsCu_2_I_3_; **c** Rb_2_CuBr_3_. Reprinted with permission from Ref. [34], Copyright 2021, Royal Society of Chemistry

Different kinds of crystal structures determine different electronic properties of materials [32]. Copper elements of different valencies combine with different halogen atoms to form various crystal structures. The crystal structures of copper-based metal halides have been studied from as early as 2004 [33]. Figure 1 shows the schematic crystal structures of three typical all-inorganic copper-based metal halides [34]. To be specific, Cs_3_Cu_2_I_5_ belongs to a typical 0D crystal structure (Fig. 1a). Two types of Cu^+^ sites, trihedral site and tetrahedral site, are present in this crystal structure. Each site is composed of [Cu_2_I_5_]^3−^ which is separated by Cs^**+**^ to form the 0D crystal structure [31]. It is worth noting that there are differences in the space group of Cs_3_Cu_2_X_5_ (X = Cl, Br and I), with Cs_3_Cu_2_Cl_5_ belonging to *Cmcm* and the remaining two belonging to *Pmpm* [35]. On the other hand, CsCu_2_I_3_ is a typical one-dimensional (1D) crystal structure (Fig. 1b). It belongs to the *Cmcm* space group. Cu^+^ combines with the surrounding I^−^ to form [Cu_2_I_3_]^−^ tetrahedra. The tetrahedra are separated by Cs^+^, and each tetrahedron extends in a co-edge manner to form 1D [Cu_2_I_3_]^−^ anionic chains [36]. Besides, Rb_2_CuBr_3_ also belongs to 1D crystal structure but in the space group *Pnma* (Fig. 1c). The [CuBr_3_]^2−^ tetrahedra separated by Rb^+^ extends in a co-angular manner to form one-dimensional long chains of anions [CuBr_3_]^2−^ [37].

### Morphology

In recent years, as the understanding of copper-based metal halides has improved, copper-based metal halides with different morphological dimensions have been successfully prepared [19, 32, 38]. It can be mainly divided into single crystals, polycrystalline powder, thin film, and nanocrystals. Materials with different morphologies can be obtained by different ingenious preparation methods [20, 39, 40].

Single crystals with a certain size can be prepared by antisolvent vapor-assisted crystallization and inverse temperature crystallization. The principle of antisolvent vapor-assisted crystallization is based on the different solubility of metal halide precursors in various solvents to grow single crystals. The slow diffusion of antisolvent vapors into the saturated precursor solution contributes to the slow growth of single crystals, but this method has a long growth time cycle. If the solubility of metal halides is low at higher temperatures, the inverse temperature crystallization method can also be adopted to grow single crystals. This method is simpler and faster than the antisolvent vapor-assisted crystallization method.

Polycrystalline powders can be synthesized by high-temperature sintering techniques and ball milling methods. In both methods, the stoichiometric ratios between the reaction materials can be adjusted to obtain products of different components and the synthetic yields are much higher than those of other methods. However, the ease of reaction of the raw materials is highly required. In general, the easier the reaction between the raw materials, the more suitable these two methods are.

Spin coating is the classic and most commonly used preparation method for thin films. The introduction of anti-solvent engineering in the process of film spin coating has greatly improved film quality. Nanocrystals can be synthesized by hot injection method and antisolvent recrystallization. In the hot injection method, different sizes of nanocrystals can be prepared by adjusting the temperature. The size of nanocrystals affects the optical properties of the material, and there is a big difference in the optical properties between materials with different sizes of nanocrystals. This is one of the advantages of quantum dot materials.

**Fig. 2:**
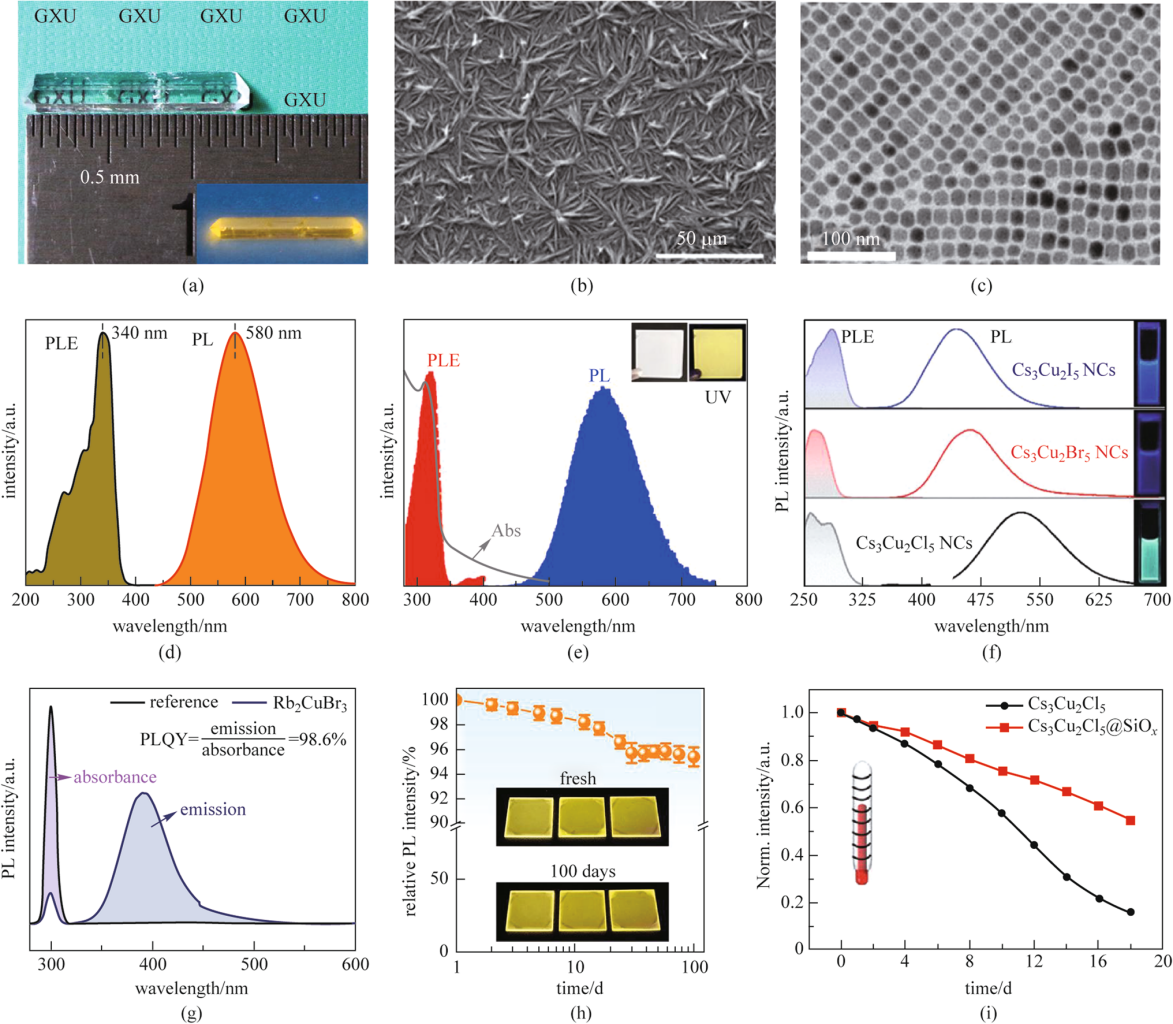
**a** Photographs of CsCu_2_I_3_ single crystals under ambient light and 365 nm UV light (inset). Reprinted with permission from Ref. [44], Copyright 2021, ELSEVIER; **b** SEM image of CsCu_2_I_3_ film. Reprinted with permission from Ref. [46], Copyright 2020, American Chemical Society; **c** TEM image of Cs_3_Cu_2_I_5_ nanocrystal. Reprinted with permission from Ref. [52], Copyright 2020, WILEY–VCH Verlag GmbH; **d** PLE and PL spectrum of CsCu_2_I_3_ single crystals. Reprinted with permission from Ref. [44], Copyright 2021, ELSEVIER; **e** Light absorption (gray), PLE (red), and PL (blue) spectrum of the CsCu_2_I_3_ film. Insets present the bright-field (left) and fluorescence photographs (right). Reprinted with permission from Ref. [46], Copyright 2020, American Chemical Society; **f** PLE and PL spectrum of Cs_3_Cu_2_X_5_ nanocrystal solutions. Right insets show the photographs of Cs_3_Cu_2_X_5_ nanocrystal solutions under 254 nm UV light. Reprinted with permission from Ref. [52], Copyright 2020, Wiley–VCH Verlag GmbH; **g** PLQY spectrum of Rb_2_CuBr_3_ crystals. Reprinted with permission from Ref. [37], Copyright 2019, WILEY–VCH Verlag GmbH; **h** PL intensity evolution of the CsCu_2_I_3_ thin films measured at different storage periods in ambient air. The insets present the photographs of the CsCu_2_I_3_ thin films under UV light excitation before and after storage for 100 days. Reprinted with permission from Ref. [47], Copyright 2020, American Chemical Society; **i** Normalized PL intensity of Cs_3_Cu_2_Cl_5_ nanocrystals and Cs_3_Cu_2_Cl_5_@SiO_*x*_ nanocrystals during a heat treatment at 80 °C. Reprinted with permission from Ref. [53], Copyright 2021, WILEY–VCH Verlag GmbH

Copper-based metal halides with different morphologies have different physical properties and different applications, as shown in Fig. 2, so their development status will be presented respectively.

#### Single crystals

In single crystal materials, the crystal cells are regularly and periodically arranged in three-dimensional space, and the entire crystal exhibits a long-range ordered nature in space. So, single crystal material has many excellent physical properties, such as high carrier mobility, low defect density, good environmental stability. Cs_3_Cu_2_X_5_ single crystals with 0D crystal structure have shown excellent optical properties and have been intensively studied. In 2018, Jun et al. [31] reported for the first time that a Cs_3_Cu_2_I_5_ single crystal was prepared by vapor antisolvent method. It was shown to have a photoluminescence (PL) peak at 445 nm with a high PLQY of 90%. The high PLQY means it has potential applications in the field of LEDs or scintillators. In 2020, Zhang et al. [41] successfully prepared Cs_3_Cu_2_I_5_ single crystals with millimeter size by the room temperature solvent evaporation crystallization method. In the same year, Lin et al. [42] prepared Cs_3_Cu_2_I_5_ single crystals with PLQY ≈ 100% by the antisolvent vapor-assisted crystallization method. They suggested that the Cs_3_Cu_2_I_5_ single crystal had a near-uniform PLQY thanks to its unique 0D crystal structure. Due to the excellent PLQY performance, they applied the material to LEDs. In 2021, Zhou et al*.* [43] prepared Cs_3_Cu_2_X_5_ (X = Cl, Br or mixed Br/Cl) single crystals by the rapid cooling crystallization method. The PLQY of these materials is ~ 100% at 525 nm, and 27% at 462 nm for X = Cl and Br respectively. They further investigated the effect of different halogen combinations on the luminescence properties of the material and found that the tunability of the luminescence wavelength can be achieved by changing the ratio of Cl^−^ to Br^−^.

In addition, CsCu_2_X_3_ and A_2_CuX_3_ (A = Rb or K, X = Cl or Br) with one-dimensional electronic structures have also attracted attention from scientists. In 2019, Lin et al*.* [36] successfully prepared CsCu_2_I_3_ single crystals with a size of 10 mm × 1.5 mm by the antisolvent infiltration method and applied them to white LEDs. It was shown to have a PL peak at 568 nm with a PLQY of 15.7%. In 2021, Mo et al*.* [44] have synthesized and demonstrated CsCu_2_I_3_ single crystals with lengths as long as 13 mm by using the inverse temperature crystallization method, as shown in Fig. 2a. The crystals showed a large Stokes shift with a photoluminescence excitation (PLE) peak at 340 nm and a PL peak at 580 nm (Fig. 2d). They used oleic acid as an additive in the process of crystal growth to increase PLQY to 50% for the first time. Replacement A-site ions have been demonstrated and widely used to improve the optical properties and the environmental stability of the material. By comparison, it is interesting to note that the PLQY of Cs_3_Cu_2_I_5_, which possesses a 0D structure, is significantly higher than that of CsCu_2_I_3_, which possesses a 1D structure. The reason is that the exciton binding energy of 0D Cs_3_Cu_2_I_5_ is much higher than that of 1D CsCu_2_I_3_, and the exciton binding energy affects the PL performance of the material at room temperature. In 2019, Yang et al*.* [37] successfully prepared Rb_2_CuBr_3_ single crystals by the slow cooling method. Rb_2_CuBr_3_ exhibits superior optical properties to CsCu_2_I_3_. It was shown to have a PL peak at 385 nm with a PLQY of 98.6%, as shown in Fig. 2g. They also demonstrated application of this single crystal in X-ray scintillators and showed superior light yield (the light yield of ~ 91,056 photons per MeV) compared to traditional materials. In 2020, Zhao et al*.* [45] prepared Rb_2_CuCl_3_ single crystals using the slow cooling method. Rb_2_CuCl_3_ exhibits violet emission at 397 nm with a PLQY of 99.4% and has a light yield of 16,600 photons per MeV. The material shows its potential applications in the field of high-energy X-ray detection.

Although the single crystal has many excellent physical properties, its complicated preparation process and the impossibility of preparing large areas limit its development. Therefore, the search for a simple and high-quality preparation method is a huge problem for copper-based metal halides.

#### Thin film and polycrystalline powder

The physical properties of thin films are not as good as those of single crystals. However, the preparation cycle of the thin film is shorter than that of the single crystals and the thin film can be prepared in a large area and are flexible. In 2020, Yang et al*.* [46] successfully prepared CsCu_2_I_3_ thin films by the antisolvent-assisted crystallization method. From the SEM image (Fig. 2a), it is clear that the quality of CsCu_2_I_3_ film needs to be further optimized. It was shown to have a PL peak at 580 nm with a PLQY of 12.3%, as shown in Fig. 2e. In the same year, Ma et al*.* [47] prepared CsCu_2_I_3_ thin films by the spin-coating method and applied them to yellow LEDs. The PLQY of the thin film was increased to 20.6% by using anti-solvent engineering. The film showed excellent stability against oxygen, moisture, and heat, as shown in Fig. 2h.

The preparation of copper-based metal halides by solution spin-coating faces a serious problem, namely the insolubility of the raw material and in turn the poor film quality. The quality of the precursor solution largely determines the quality of the film during the preparation of the film by the solution method. To avoid this problem, some groups have turned their attention to solid-phase reaction methods that do not require solutions. The solid-phase reaction completely solves the insolubility problem of raw materials and the preparation process is simpler. In 2019, Roccanova et al*.* [48] successfully synthesized Cs_3_Cu_2_Br_5–*x*_I_*x*_ (0 ≤ *x* ≤ 5) polycrystalline powder by a solid-phase reaction. The PLQY increase linearly with *x* from 50.1% for Cs_3_Cu_2_Br_5_ to 98.7% for Cs_3_Cu_2_I_5_. In 2020, Grandhi et al*.* [35] prepared high-quality phase-pure Cs_3_Cu_2_X_5_ polycrystalline powders by an all-solid-state mechanochemical synthesis method. They focused on the structural transformation of the raw materials with different stoichiometric ratios in solid-phase reactions. For example, the amount of CsI affects the phase transition between Cs_3_Cu_2_I_5_ and CsCu_2_I_3_. The chemical reaction of 4 mol of CsI with 1 mol of Cs_3_Cu_2_I_5_ produces 3 mol of CsCu_2_I_3_. In this process, the isolated [Cu_2_I_5_]^3−^ units of Cs_3_Cu_2_I_5_ are transformed into double chains of [Cu_2_I_3_]^−^. This transformation process can be reversed by the addition of CsI to CsCu_2_I_3_. The transformation of the crystal structure from 1D to 0D causes a great change in the luminescence performance, which can be achieved by electronic structure design. In 2020, Xie et al. [49] used the ball milling method to synthesize Cs_3_Cu_2_X_5_ with PLQY up to 60%. The blue emissive Cs_3_Cu_2_I_5_ and green emissive Cs_3_Cu_2_Cl_5_ polycrystalline powders obtained have good thermal stability and photostability.

Although copper-based metal halide thin films and polycrystalline powders have achieved relatively good research results, they still face some serious problems, such as non-uniform films, and poor film quality.

#### Nanocrystals

In 2019, Cheng et al. [50] successfully prepared 1D CsCu_2_I_3_ nanorods and 0D Cs_3_Cu_2_I_5_ nanocrystals by the hot injection method. They found that the reaction temperature was an important factor affecting the final product in the preparation. When the reaction temperature was 110 °C, the end product was CsCu_2_I_3_, and when the reaction temperature was 70 °C, the end product was Cs_3_Cu_2_I_5_. They also found that CsCu_2_I_3_ was not stable in solution and tended to form nanorods. In 2020, Li et al. [51] synthesized Cs_3_Cu_2_X_5_ (X = I, Br/I, Br, Br/Cl and Cl) nanocrystals at room temperature by the antisolvent method. These nanocrystals have uniform sizes of less than 10 nm in diameter and show excellent optical properties, including composition-tuned emission spectrum over the spectral region of 440 − 530 nm, and with high PLQY of ∼100%, 20%, and 30% for X = Cl, Br, and I, respectively. In 2020, Luo et al. [52] prepared Cs_3_Cu_2_X_5_ nanocrystals by the hot-injection method, as shown in Fig. 2c. These Cs_3_Cu_2_X_5_ nanocrystals exhibited broadband blue-green photoluminescence emissions in the range of 445–527 nm with large Stokes shifts, as shown in Fig. 2f. It is worth paying attention to the PL emissions of Cs_3_Cu_2_X_5_ nanocrystals, which are quite different from those of typical CsPbX_3_ nanocrystals. CsPbX_3_ nanocrystals show a blueshift via successive substitution of X halogen ion from I^−^ to Br^−^, and then to Cl^−^, while Cs_3_Cu_2_X_5_ show redshift by successive substitution of X-halogen ions from I^−^ to Br^−^, and then to Cl^−^. The reason for this phenomenon is the unique self-trapped exciton (STE) luminescence behavior of copper-based metal halides. For STE emission, the emission energy is not only related to the bandgap, but also depends on the exciton binding energy, lattice distortion energy and self-trapping energy. In addition, Cs_3_Cu_2_Cl_5_ nanocrystals have the relatively best luminescence performance with a PLQY of 48.7%. Cs_3_Cu_2_I_5_ nanocrystals exhibit considerable air stability over 45 days.

Among the studies on copper-based metal halides, Cs_3_Cu_2_Cl_5_ nanocrystals have attracted much attention due to their excellent luminescence properties. To further improve their luminescence properties and stability, Zhao et al. [53] optimized Cs_3_Cu_2_Cl_5_ nanocrystals by encapsulating. They coated Cs_3_Cu_2_Cl_5_ nanocrystals with SiO_*x*_ shells to increase the PLQY of Cs_3_Cu_2_Cl_5_ nanocrystals to 76%. Due to the presence of SiO_*x*_ shells, Cs_3_Cu_2_Cl_5_ nanocrystals have improved stability against water, moisture, and heat. Cs_3_Cu_2_Cl_5_@SiO_*x*_ nanocrystals show potential for luminescence applications due to their excellent luminescence properties and environmental stability, as shown in Fig. 2i.

For a better comparison, the differences between the optical properties of copper-based metal halides with different morphologies are summarized in Table 1.

**Table 1:** Summary of optical parameters of copper-based metal halides

Formula	Morphology	Method	PL/nm	PLQY/%	Ref.
Cs_3_Cu_2_I_5_	Single crystal	Vapor antisolvent method	445	90	[31]
Cs_3_Cu_2_I_5_	Single crystal	Solvent evaporation crystallization method	442	89	[41]
Cs_3_Cu_2_I_5_	Single crystal	Antisolvent vapor-assisted crystallization method	443	~ 100	[42]
Cs_3_Cu_2_Br_5_	Single crystal	Rapid cooling crystallization method	462	27	[43]
Cs_3_Cu_2_Cl_5_	Single crystal		525	~ 100	
CsCu_2_I_3_	Single crystal	Antisolvent infiltration method	568	15.7	[36]
CsCu_2_I_3_	Single crystal	Inverse temperature crystallization method	580	50	[44]
Rb_2_CuBr_3_	Single crystal	Slow cooling method	385	98.6	[37]
Rb_2_CuCl_3_	Single crystal	Slow cooling method	397	99.4	[45]
CsCu_2_I_3_	Film	Antisolvent-assisted crystallization method	580	12.3	[46]
CsCu_2_I_3_	Film	Spin-coating method	548	20.6	[47]
Cs_3_Cu_2_I_5_	Powder	Solid-phase reaction	443	98.7	[48]
Cs_3_Cu_2_Br_5_	Powder		454	50.1	
Cs_3_Cu_2_I_5_	Powder	All-solid-state mechanochemical synthesis method	440	62	[35]
Cs_3_Cu_2_Br_5_	Powder		460	10	
Cs_3_Cu_2_Cl_5_	Powder		525	60	
Cs_3_Cu_2_I_5_	Powder	Ball milling method	440	60	[49]
Cs_3_Cu_2_Cl_5_	Powder		510	53	
CsCu_2_I_3_	Nanorods	Hot injection method	553	5	[50]
Cs_3_Cu_2_I_5_	Nanocrystals		441	67	
Cs_3_Cu_2_I_5_	Nanocrystals	Room temperature antisolvent method	443	30	[51]
Cs_3_Cu_2_Br_5_	Nanocrystals		458	20	
Cs_3_Cu_2_Cl_5_	Nanocrystals		521	~ 100	
Cs_3_Cu_2_I_5_	Nanocrystals	Hot injection method	445	29.2	[52]
Cs_3_Cu_2_Br_5_	Nanocrystals		461	16.9	
Cs_3_Cu_2_Cl_5_	Nanocrystals		527	48.7	
Cs_3_Cu_2_Cl_5_@SiO_*x*_	Nanocrystals	Hot-injection method	523	76	[53]

## Photophysics and optical properties

### Photophysics

**Fig. 3:**
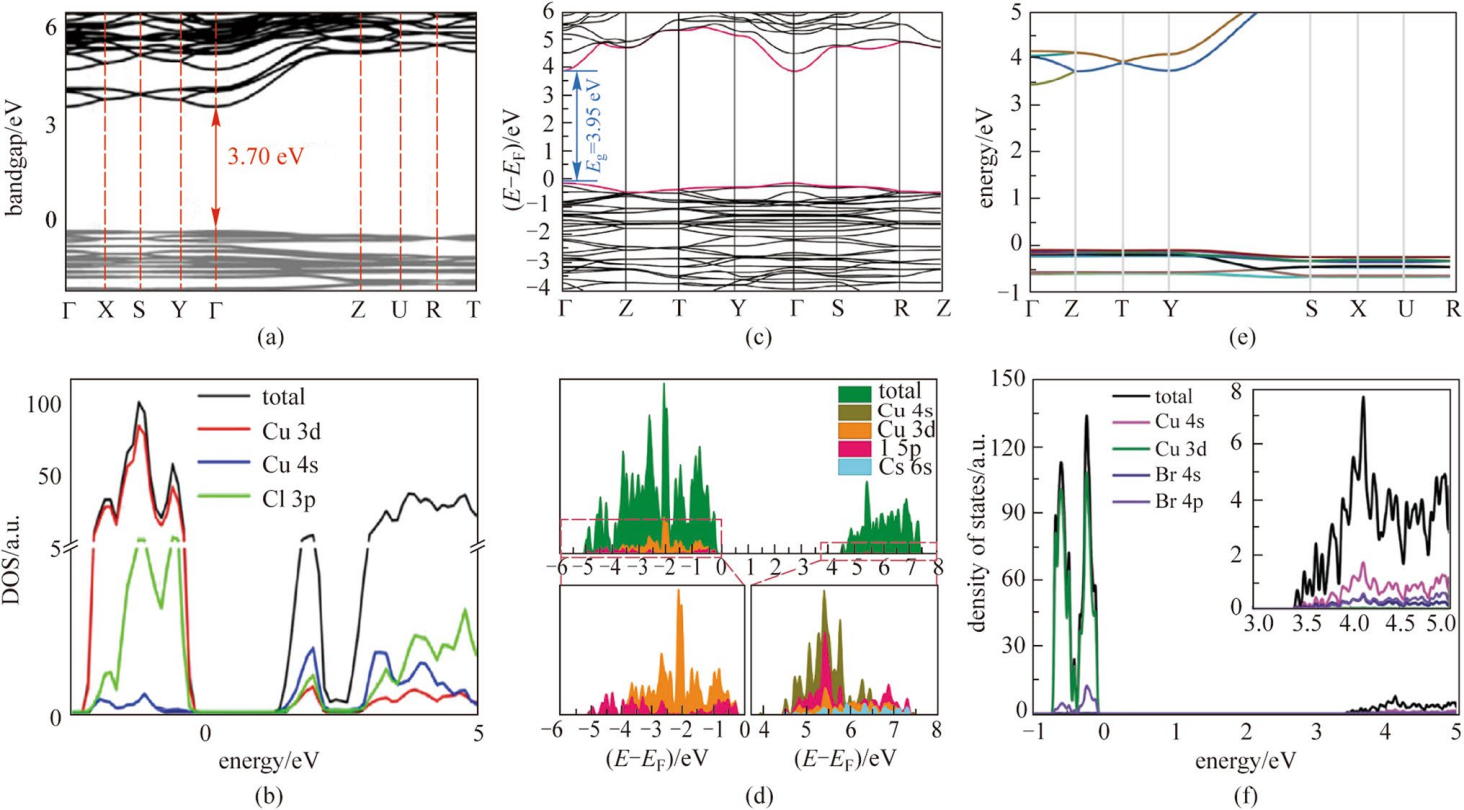
Electronic properties of Cs_3_Cu_2_Cl_5_: **a** Electronic band structure, **b** PDOSs. Reprinted with permission from Ref. [54], Copyright 2020, WILEY–VCH Verlag GmbH; Electronic properties of CsCu_2_Cl_3_: **c** Electronic band structure, **d** PDOSs. Reprinted with permission from Ref. [36], Copyright 2021, ELSEVIER; Electronic properties of Rb_2_CuBr_3_: **e** Electronic band structure, **f** PDOSs. Reprinted with permission from Ref. [37], Copyright 2019, WILEY–VCH Verlag GmbH

Most properties (such as charge transfer and optical properties) of metal halide systems are determined by their electronic structures, including the spatial and energy distribution of electrons. To date, the band structures, the projected density of states (PDOSs), and many other electronic properties of copper-based metal halides have been studied extensively. The typical electronic structures and PDOSs of copper-based metal halides are shown in Fig. 3.

Cs_3_Cu_2_Cl_5_ is a direct bandgap semiconductor with both the conduction band minimum (CBM) and valence band maximum (VBM) being located at the Γ point, as shown in Fig. 3a. The theoretical calculation shows that the bandgap is 3.70 eV, which is not much different from the experimental measurement of 3.65 eV. From knowledge of PDOSs we can understand the electronic orbital structure of Cs_3_Cu_2_Cl_5_ more intuitively and deeply. We can see that the VBM is mainly composed of Cu 3d and Cl 4p orbitals, whereas the CBM consists of both Cu 4 s and Cl 3p orbitals [54]. Cs is not involved in the composition of these electron orbitals, and electrons and holes are mainly concentrated on Cu and Cl, as shown in Fig. 3b. This corresponds to the crystal structure of Cs_3_Cu_2_Cl_5_. It is noteworthy that the exciton binding energy of copper-based metal halides is much greater than that of lead-based metal halides. The exciton binding energy of Cs_3_Cu_2_Cl_5_ is about 553.55 meV resulting in higher stability and enhanced PL emission at room temperature. The Huang-Rhys factor of the Cs_3_Cu_2_Cl_5_ films is 26.21 which indicates that Cs_3_Cu_2_Cl_5_ has a soft crystal lattice, where the excitons are easily self-trapped and generate STE emissions [55]. Figure 3c, d shows the first-principles calculation results of CsCu_2_I_3_. In the energy band diagram, CsCu_2_I_3_ has a direct band gap and a theoretically calculated band gap of 2.05 eV, with a low density of states at CBM and a flat density of states at VBM. From the state density, the VBM is mainly composed of Cu 3d and I 5p orbitals, while the CBM mainly contains Cu 4 s and I 5p orbitals. The Cs contribution to VBM and CBM is too small to be considered [36]. The exciton binding energy of CsCu_2_I_3_ is about 346.23 meV and the Huang-Rhys factor of the CsCu_2_I_3_ is 19.84 [36]. Such high exciton binding energy comes from its unique one-dimensional electronic structure. The Cu–I octahedron providing electronic state is strongly isolated by Cs atoms along 1D direction, which strengthens the localization of exciton. The electronic structure of Rb_2_CuBr_3_ is similar to that of CsCu2I3. Figure 3e, f shows the electronic energy band structure and the PDOSs of Rb_2_CuBr_3_. It is a direct band gap semiconductor with a bandgap of 3.51 eV at the Γ point. It is evident from the energy band diagram that the density of states at the CBM is low, while it is relatively flat at the VBM. The PDOSs indicates that the CBM of Rb_2_CuBr_3_ is primarily composed of Cu 4 s, Br 4 s, and Br 4p orbitals, whereas the VBM consists of Br 4p and Cu 3d orbitals, and Rb does not contribute to CBM or VBM [37]. The exciton binding energy of Rb_2_CuBr_3_ is about 758.87 meV and the Huang-Rhys factor of the Rb_2_CuBr_3_ films is 37.17 [37].

### Optical properties

Copper-based metal halides are dominated by 0D and 1D crystal structures, and their low-dimensional crystal structures possess strong electron–phonon coupling. In addition, the crystal structure is easily distorted under external stimulation, such as UV and X-ray excitation. These special crystal structure characteristics contribute to the unique photophysics/optical properties of copper-based metal halides. Most copper-based metal halides were reported to exhibit wide full width at half maxima (FWHM), large Stokes shifts as well as long lifetimes, which can be attributed to STE emission.

**Fig. 4:**
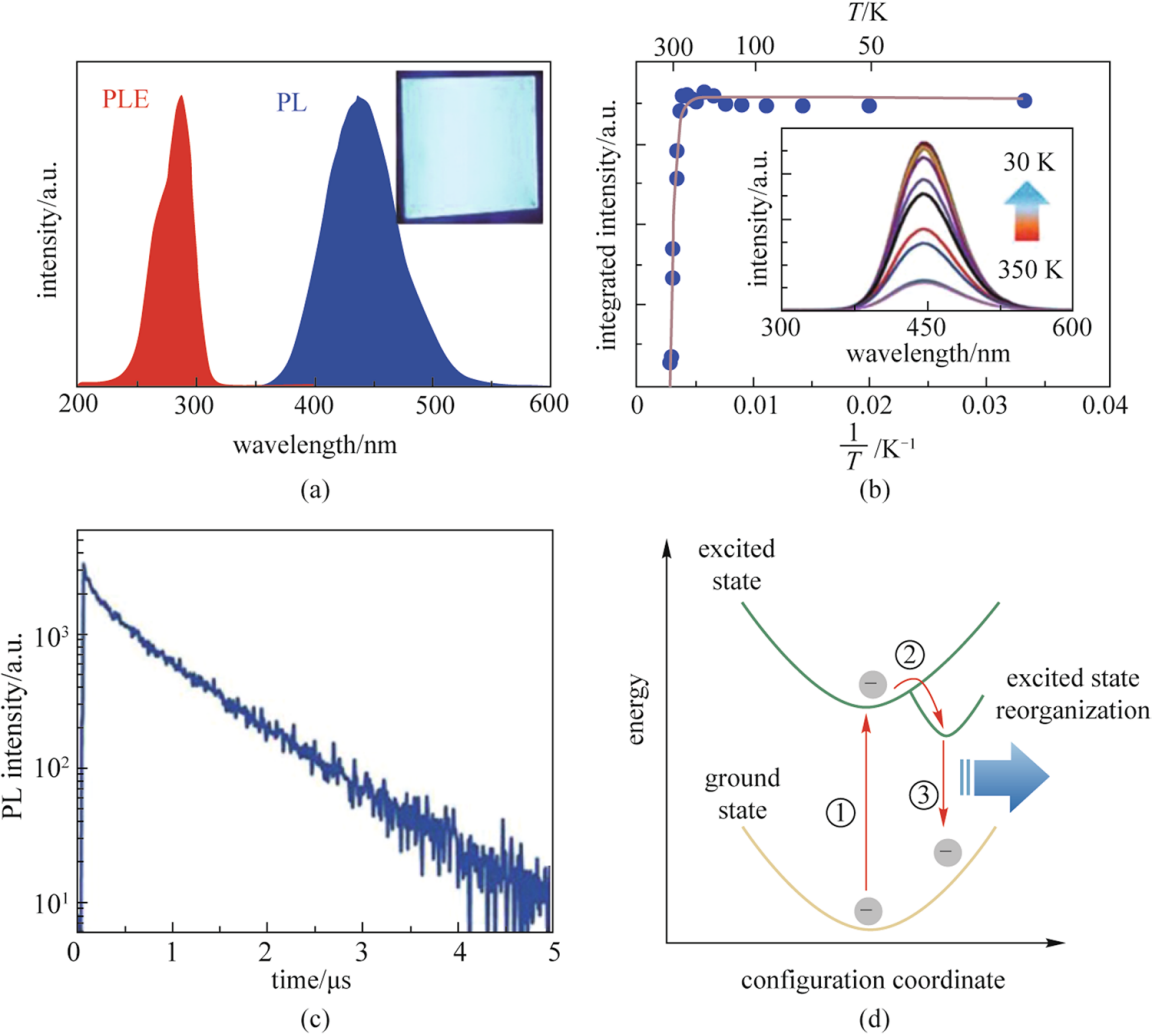
Optophysical properties of Cs_3_Cu_2_I_5_: **a** PL and PLE spectrum of the Cs_3_Cu_2_I_5_ thin film; **b** Integrated PL intensity as a function of reciprocal temperature from 30 to 350 K. The inset of **b** shows the temperature-dependent PL spectrum; **c** Time-resolved PL decay curve at room temperature; **d** Mechanism of configuration coordinate for the excited-state reorganization. Reprinted with permission from Ref. [31], Copyright 2018, WILEY–VCH Verlag GmbH

As a classical phenomenon originating from soft lattices and strong electron–phonon coupling, STE luminescence has been intensively studied in many fields. Under the effect of photoexcitation, the strong electron–phonon coupling leads to lattice distortion. Due to the lattice distortion, electrons and holes are separated in space to form a stable self-trapped exciton state. This self-trapped exciton state has a smaller band gap and stronger localization effect than the original state. Finally, the material will exhibit optical properties of broad-spectrum, large Stokes shift and long lifetime (Fig. 4) [56, 57]. The contribution of A-site ions to the material properties was found to be very small in the studies of the crystal structure and energy bands of copper-based metal halides. Therefore, the optical properties of copper-based metal halides are mainly influenced by the interaction between copper atoms and halogens. The optical properties of both Cs_3_Cu_2_X_5_, which has a 0D structure, and CsCu_2_X_3_ and A_2_CuX_3_, which have a 1D structure, can be attributed to STE. Next, Cs_3_Cu_2_I_5_ is used as an example to deeply understand the principle of STE. The PLE and PL peak wavelengths are 290 and 445 nm, respectively, as shown in Fig. 4a. A large Stokes shift of ~ 155 nm is similar to those of other 0D metal halides. In addition, the exciton binding energy evaluated for Cs_3_Cu_2_I_5_ was ~ 490 meV and the exciton lifetime of Cs_3_Cu_2_I_5_ was ~ 464 ns, which is much larger than that of the 3D metal halides, as shown in Fig. 4b, c. The main reason for these properties is the excited-state structural reorganization. Under photoexcitation conditions, the Cu(I)-3d10 electron orbital is distorted to form the Cu(II)-3d9. The change of electron orbital configurations leads to the reorganization of the excited state structure, while the Stokes shift depends mainly on the energy difference between the excited states, i.e., between Cu(I)-3d10 and Cu(II)-3d9. In addition, the strong electron–phonon coupling leads to the deformation of the lattice, resulting in the spatial separation of electrons from holes. Therefore, the carrier recombination process is spatially suppressed leading to a long exciton lifetime [31]. In conclusion, we can understand the STE luminescence mechanism from the molecular orbital theory, as shown in Fig. 4d. When the material is in an excited state, Cu(I)-3d10 is deformed into Cu(II)-3d9, which produces the Jahn–Teller effect and changes the energy level distribution of the excited state.

## Applications

### Photodetectors

**Fig. 5:**
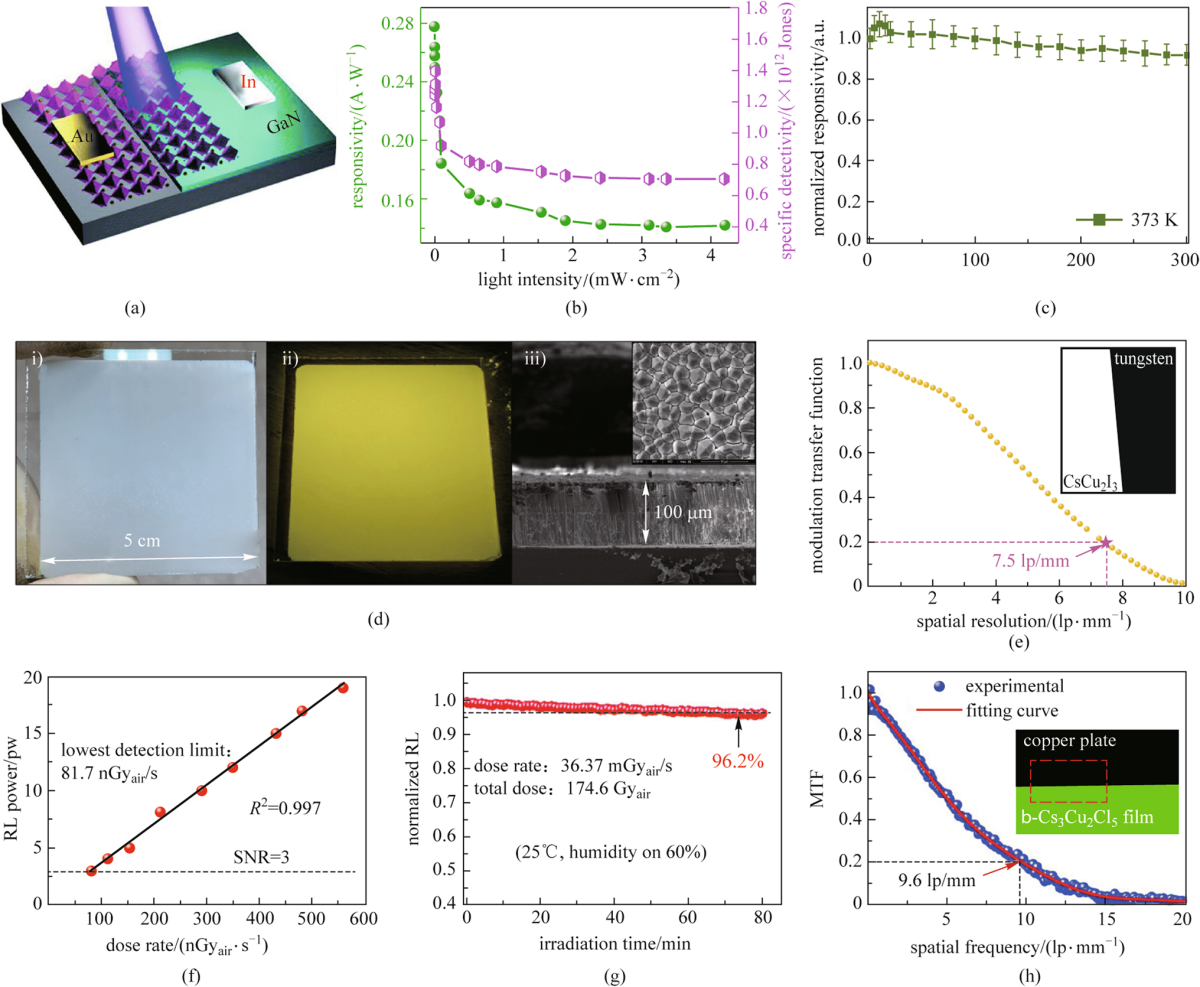
**a** Schematic illustration of the Cs_3_Cu_2_I_5_/GaN heterojunction device; **b** Responsivity and specific detectivity of the photodetector versus light intensity; **c** Responsivity evolution of the heterojunction photodetector monitored at 373 K in ambient air. **a**–**c** Reprinted with permission from Ref. [59], Copyright 2020, Royal Society of Chemistry; **d** CsCu_2_I_3_ film for the X-ray imaging application: (i) Optical photograph, (ii) photoluminescent photograph, and (iii) SEM image of oriented structured CsCu_2_I_3_ thick film; **e** Modulation transfer function (MTF) of the CsCu_2_I_3_ detector, measured by the slanted-edge method (inset). **d**, **e** Reprinted with permission from Ref. [62], Copyright 2021, American Chemical Society; **f** Lowest detection limit of Cs_3_Cu_2_Cl_5_ films at signal noise ratio (SNR) of 3; **g** Operational stability of Cs_3_Cu_2_Cl_5_ films by continuous X-ray irradiation; **h** MTF of Cs_3_Cu_2_Cl_5_ films. **f**–**h** Reprinted with permission from Ref. [64], Copyright 2021, Royal Society of Chemistry

With the various needs of society, high performance UV detectors is becoming more and more in demand. Owing to their excellent optical properties in UV regions, copper-based metal halides have gained wide application in the field of UV detection as shown in Fig. 5.

In 2019, Zhang et al. [58] first reported a deep-ultraviolet photodetector made up of Cs_3_Cu_2_I_5_. They prepared a planar structured UV detector with Cs_3_Cu_2_I_5_ film deposited on ITO glass by the method of slow vapor saturation of an antisolvent. The UV detector shows high sensitivity to deep UV light at 265 nm. Specifically, at 1 V, the responsivity, detectivity, and external quantum efficiency (EQE) were 64.9 mA/W, 6.9 × 10^11^ Jones, and 0.3%, respectively. In 2020, Li et al. [59, 60] successfully prepared Cs_3_Cu_2_I_5_/GaN heterojunctions (Fig. 5a) to achieve selective detection in the 300–370 nm. The photodetectors demonstrate a high responsivity of 0.28 A/W, a specific detectivity of 1.4 × 10^12^ Jones (Fig. 5b), an on/off photocurrent ratio of 1.2 × 10^5^, and fast response speeds of 95 (rise time)/130 (fall time) μs under UV light excitation (320 nm). In addition, the device exhibits excellent operating stability in open air environments, as shown in Fig. 5c. In the same year, they also discovered that CsCu_2_I_3_ nanowires possess polarized UV detection capabilities due to the intrinsic anisotropy of their asymmetric structure and the anisotropy of their external morphology. They prepared CsCu_2_I_3_ nanowires as polarization-sensitive UV detectors, and the devices obtained a photoresponsivity of 32.2 A/W, a specific detectivity 1.89 × 10^12^ Jones, and response speeds of 6.94 (rise time)/214 (fall time) μs. They also prepared devices with flexible substrates, which have good flexibility and stability, with almost no degradation of performance after 1000 bending cycles. In 2021, Ma et al. [61] prepared a Cs_3_Cu_2_I_5_/β-Ga_2_O_3_ heterojunction and applied it to a solar-blind UV photodetector. This photodetector exhibits a low dark current of 1.2 pA, a high photoresponsivity of 2.3 mA/W, and a high on/off ratio of ∼5.1 × 10^4^ at zero bias, under 265 nm light illumination.

Overall, copper-based metal halides are promising candidates for low-cost, high-performance UV photodetectors.

### X-ray scintillators

All-inorganic copper-based metal halides are also attracting attention in the field of scintillators due to their large Stokes shift and high PLQY as shown in Fig. 5.

In 2019, Yang et al. [37] used Rb_2_CuBr_3_ as an X-ray scintillator, which exhibited excellent performance. The light yield of 91,056 photons per MeV was much higher than that of traditional scintillators. In 2020, Zhao et al*.* [45] studied the photovoltaic properties of Rb_2_CuCl_3_ materials and applied them to the field of X-ray indirect detection. Rb_2_CuCl_3_ demonstrated an appreciable light yield of 16,600 photons per MeV and large and linear scintillation response within a range from 48.6 nGy_air_/s to 15.7 μGy_air_/s. In 2021, Zhang et al*.* [62] prepared a large-area (25 cm^2^) CsCu_2_I_3_ scintillator film with good scintillation properties by oriented structural design as shown in Fig. 5d. CsCu_2_I_3_ films with a columnar crystal shape were found to effectively reduce light scattering and improve X-ray imaging quality. This CsCu_2_I_3_ scintillator achieved a high spatial resolution of 7.5 lp/mm in X-ray imaging as shown in Fig. 5e. There are two factors that affect the quality of X-ray imaging: light yield and light scattering. The light yield depends on how well the material itself responds to X-rays. The effect of light scattering can be largely reduced by structural design. In 2021, Zhao et al. [63] prepared a high-performance scintillator by combining Cs_3_Cu_2_I_5_ with anodic aluminum oxide (AAO). As Cs_3_Cu_2_I_5_ was confined in a hollow columnar structure formed by the AAO, light scattering was significantly reduced and the imaging quality was further improved. The Cs_3_Cu_2_I_5_–AAO scintillator demonstrated high spatial resolution (10.4 lp/mm at modulation transfer function (MTF) = 0.2). In 2021, Zhou et al*.* [64] prepared Cs_3_Cu_2_Cl_5_ nanocrystal thin films by a spin coating method. The scintillator film had a light yield of 34,000 ± 4000 photons per MeV, a spatial resolution of 9.6 lp/mm in X-ray imaging (Fig. 5h), and a minimum detection limit of 81.7 nGy_air_/s (Fig. 5f). In addition, the scintillator exhibited excellent environmental and irradiation stability as shown in Fig. 5g. Flexible X-ray scintillators are a key problem in the field of X-ray detection. In the medical field, flexible scintillators allow for more detailed and clear imaging, enabling doctors to make more accurate judgments about the pathological tissue. In 2022, Han et al*.* [55] tuned the crystal structure and optical properties of Cs_3_Cu_2_Cl_5_ by doping K^+^ and enhanced the PLQY of Cs_3_Cu_2_Cl_5_ nanosheet to 81.39%. They prepared large-area flexible scintillator films by combining this doped nanosheet with polystyrene. The flexible scintillator film exhibited a very sensitive scintillation response to X-ray signals within 20–160 keV. This work provides a new idea for future preparation and research of flexible scintillator materials.

Light yield is a particularly important parameter in evaluating the performance of scintillator materials. Metal ion doping has shown significant advantages in increasing the light yield of scintillators. Wang et al. [65] optimized the optical properties of Cs_3_Cu_2_I_5_ by doping In^+^ and prepared Cs_3_Cu_2_I_5_:In^+^ single crystals by the vertical Bridgman method. In^+^ doping enhanced the PLQY of Cs_3_Cu_2_I_5_ from 68.1 to 88.4%. Benefiting from the higher PLQY, Cs_3_Cu_2_I_5_:In^+^ can achieve a superior light yield of 53,000 photons per MeV, which is comparable to commercial CsI:Tl single crystals (54,000 photons per MeV). Li et al*.* [66] prepared Cs_3_Cu_2_I_5_:Mn^+^ scintillator material, and the light yield was enhanced to 67,000 photons per MeV due to the introduction of new luminescence centers by Mn^+^ doping, which reduced the non-radiative complex of Cs_3_Cu_2_I_5_ and overcame the temperature quenching effect of the intrinsic material. Rare earth elements have been intensively investigated in doping engineering. Cheng et al*.* [67] prepared the Tl-doped Cs_3_Cu_2_I_5_ crystals with excellent optical properties, exhibiting a high PLQY of 79.2% and the light yield of 87,000 photons per MeV. Optical yield largely determines the imaging quality of scintillator materials. Copper-based metal halides show excellent optical yield performance with the optimization of ion doping engineering, proving their potential advantages in the field of scintillator materials.

Although copper-based metal halides have achieved some success in the field of X-ray scintillators and photodetection, there are still some problems, such as the low resolution of X-ray imaging, and difficulty in achieving large-area high-quality flexible films.

## Conclusions and perspectives

We have reviewed the developments in the field of copper-based metal halides in recent years and summarized their development status and problems in terms of crystal structure/morphology, photophysics/optical properties and applications. Although copper-based metal halides have achieved excellent results in many areas, there are still many new opportunities and challenges:

1. Mechanism. Copper-based metal halides possess excellent optical properties due to their unique crystal structure and low electronic dimension. The luminescence properties of this class of materials were found to be derived from STE, and there are still many problems in the study of the luminescence principles of STE. Further studies on the relationship between photoexcitation, crystal structure changes, and excited state reorganization are still needed;
2. Stability. Copper-based metal halides are soft lattice materials that undergo lattice deformation when excited by UV light. There are relatively few studies on how the lattice deformation is restored and whether the lattice deformation affects the stability of the material. Therefore, the study of the lattice stability of copper-based metal halides is a significant direction. If the material stability problem can be solved, they will realize more in-depth applications in the field of X-ray detection;
3. Fabrication. The current research on all-inorganic copper-based metal halides is mainly focused on material preparation. The preparation cycle of single crystal materials is too long and the production efficiency is low. In addition, it is difficult to prepare thin films by a solution method due to the insolubility of raw materials, and the quality of quantum dot films needs to be further improved. The search for new preparation processes is the biggest issue at present. The vacuum evaporation method has shown great advantages with insoluble materials and the films prepared by this method are of good quality, uniformity and flatness. The raw materials of copper-based metal halides are also insoluble, so the development of a vacuum vapor deposition method for the preparation of fully inorganic copper-based metal halides is also a worthy research direction.
